# Sporadic Premature Aging in a Japanese Monkey: A Primate Model for Progeria

**DOI:** 10.1371/journal.pone.0111867

**Published:** 2014-11-03

**Authors:** Takao Oishi, Hiroo Imai, Yasuhiro Go, Masanori Imamura, Hirohisa Hirai, Masahiko Takada

**Affiliations:** 1 Systems Neuroscience Section, Primate Research Institute, Kyoto University, Inuyama, Japan; 2 Molecular Biology Section, Primate Research Institute, Kyoto University, Inuyama, Japan; 3 Department of Brain Sciences, Center for Novel Science Initiatives, National Institutes of Natural Sciences, Tokyo, Japan; 4 Department of Developmental Physiology, National Institute for Physiological Sciences, Okazaki, Japan; University of Toyama, Japan

## Abstract

In our institute, we have recently found a child Japanese monkey who is characterized by deep wrinkles of the skin and cataract of bilateral eyes. Numbers of analyses were performed to identify symptoms representing different aspects of aging. In this monkey, the cell cycle of fibroblasts at early passage was significantly extended as compared to a normal control. Moreover, both the appearance of senescent cells and the deficiency in DNA repair were observed. Also, pathological examination showed that this monkey has poikiloderma with superficial telangiectasia, and biochemical assay confirmed that levels of HbA1c and urinary hyaluronan were higher than those of other (child, adult, and aged) monkey groups. Of particular interest was that our MRI analysis revealed expansion of the cerebral sulci and lateral ventricles probably due to shrinkage of the cerebral cortex and the hippocampus. In addition, the conduction velocity of a peripheral sensory but not motor nerve was lower than in adult and child monkeys, and as low as in aged monkeys. However, we could not detect any individual-unique mutations of known genes responsible for major progeroid syndromes. The present results indicate that the monkey suffers from a kind of progeria that is not necessarily typical to human progeroid syndromes.

## Introduction

Symptoms representing various aspects of aging at an early age are characteristic of progeria in humans. Progeria is a series of very rare genetic syndromes that are related to mutations in specific genes. For example, genes responsible for Werner syndrome (WS), Rothmund-Thomson syndrome (RTS), Bloom syndrome (BS), and Hutchinson-Gilford progeria syndrome (HGPS) are RECQ3 (WRN), RECQL4, RECQ2, and LMNA, respectively [1], [2]. As summarized in Table 1, these syndromes are accompanied by common or separate physical abnormalities that can be ascribed to senile changes. An animal model is a powerful tool to investigate possible mechanisms underlying not only premature aging, but also normal aging. However, no useful animal models have so far been available. Although mice lacking the RECQL gene have been reported, their pathological changes are quite limited, probably due to a species difference between rodents and humans [3]. Thus, a primate model for progeria, which is taxonomically akin to humans and has a longer life-span than a rodent model, is needed to promote aging research. Recently, we have found a child Japanese monkey who displayed deep skin wrinkles and bilateral cataract at the age of one year. In the present study, we examined whether this monkey is actually progeroid or not from various viewpoints.

**Table 1:** Physical signs and symptoms characteristic of human progeroid syndromes and monkey N416.

Signs and symptoms	WS	RTS	HGPS	BS	N416
cataract	✓	✓			✓
abnormal glucose metabolism	✓				✓
urinary hyaluronan	✓		✓		✓
decline in proliferative potency of fibroblasts					
at early passage	✓				✓
increase in senescent fibroblast cells	(✓)	✓	✓	✓	✓
DNA repair deficiency	✓	✓		✓	✓
poikiloderma		✓		✓	✓
short statue, low bodyweight	✓	✓	✓		
facial proportion	✓	✓	✓		
loss of hair	✓	✓	✓		
facial rash		✓		✓	
intractable skin ulcers	✓				
prominent scalp vein			✓		
sun-sensitivity		✓		✓	
skeletal defects	✓	✓	✓		
abnormal dentation			✓		
characteristic voice	✓		✓	✓	
soft-tissue calcification	✓				
contracture of joints			✓		
atherosclerosis	✓		✓		
malignant tumors	✓	✓			
hypogonadism	✓	✓		✓	nt
immunodeficiency		✓		✓	nt
onset after adolescence	✓				
onset within one year of age		✓	✓		✓

BS: Bloom syndrome, HGPS: Hutchinson-Gilford progeria syndrome, nt: not tested, RTS: Rothmund-Thomson syndrome, WS: Werner syndrome, (✓): Controversial [10], [11].

## Methods

### Animals and ethics statements

All animal experiments were planned and executed in strict concordance with Guide for the Care and Use of Laboratory Animals (8th Ed, 2011, The National Academies, USA) and Guidelines for Care and Use of Nonhuman Primates (Ver. 3, 2010, Primate Research Institute, Kyoto University). The protocol was approved by the Animal Welfare and Animal Care Committee, Primate Research Institute, Kyoto University (Permission No. 2012-115). All monkeys used in this study, weighing less than 10 kg, were kept in indoor individual cages (0.89 m×0.63 m×0.82 m) of the Primate Research Institute on a 12-h on/12-h off lighting schedule. In each cage, the monkeys were given *ad libitum* access to food and water, and a chain-hung wood block as a toy. No monkeys were sacrificed. Monkey N416, a female Japanese monkey (*Macaca fuscata*) who was supposed to be progeroid, was born in an outdoor cage (5 m×5 m×2.7 m, 6–7 monkeys per cage) and moved to an indoor individual cage at the age of one year and four months to avoid progress of cataract. Unfortunately, N416 suddenly died of bloat at the age of three years.

### Measurements of cell proliferation

Cell proliferation was analyzed using fibroblast cells prepared from cultures of a tiny block of the ear skin as described previously [4]. To determine the duplication time of each cell, cultures were made using a medium of Amnio Max II Complete (Gibco BRL, USA) in a 60×15 mm petri dish for cell culture (FALCON, 353002, USA). After appropriate proliferation of cells in the primary dish culture, cells were harvested, and then cell suspension (25–30 cells/µl) was transferred to a 35×10 mm cell culture dish (Advanced glass bottom, Greiner Bio-one, 627965, Germany). One day later, recordings of the cell division were performed every five minutes with a time-lapse imaging system (Cell Observer, Carl Zeiss, Germany). The time of a cell cycle was figured out by chasing each cell division based on imaging data collected in 70-hr cultures: the time was calculated as an interval time from the first division to the second division of each cell. A total of 44 or 47 cells were analyzed in monkey N416 and a normal control, respectively. To measure the population growth activity, fibroblast cells derived from monkey N416 and normal infant and aged monkeys (all at passage 8) were seeded at 1×10^5^ cells/well in gelatinized 6-well plates with DMED containing 15% FBS, 0.1 mM non-essential amino acids, 2 mM L-glutamine, 1 mM sodium pyruvate, 0.11 mM 2-mercaptoethanol, 100 U/ml penicillin, and 100 µg/ml streptomycin. The total cell numbers in three independent wells were counted every 3 days up to day 12.

### Detection of senescent cells

Senescent cells were detected with ß-galactosidase assay using fibroblast cells prepared from monkey N416, a normal infant and a normal aged monkey. The number of passage of fibroblast cells derived from monkey N416 and the infant and the aged controls was 5, 7, and 5, respectively. Senescence-associated ß-galactosidase activity was detected by using Cellular Senescence Assay Kit (CBA-230, Cell Biolabs, USA). Cells were fixed and incubated with X-gal overnight. Five images were randomly captured (BZ-9000, Keyence, Japan) from each culture dish of the three monkeys, and the numbers of stained and unstained cells were counted.

### Quantification of DNA repair

To test DNA repair deficiency in monkey N416, we quantified DNA damage by counting apurinic/apyrimidinic sites (AP sites), which are the target of base excision repair. DNA was freshly prepared with DNeasy Blood & Tissue Kit (69504, Qiagen, USA) from fibroblast cells of the three monkeys used for the senescent cell detection. Fibroblast cells from the infant control had been divided into normal and UV-irradiated (1 minute) groups. The AP sites were labeled with DNA Damage Quantification Kit (DK02, Dojindo, Japan) and then quantified (Flex Station 3, Molecular Devices, USA).

### Pathological analyses

A small piece of skin tissue was taken from the upper arm of monkey N416 at her health check (1 year old), and histological specimens were prepared with hematoxylin-eosin (HE) staining. Immediately after her sudden death, the heart, lung, stomach, intestine, liver, kidney, and spleen were dissected and HE specimens were prepared.

### X-ray CT and MRI

Images of the head and extremities of monkey N416 and a control monkey of the same age (2 years old) were obtained with Asteion TSX-021B (Toshiba Medical Systems). The monkeys were anesthetized with ketamine hydrochloride (5 mg/kg, i.m.) and medetomidine (100 µg/kg, i.m.), followed by head fixation with a stereotaxic apparatus [5]. Three-dimensional reconstruction was done with VirtualPlace (Aze).

T1-weighed MRIs of monkey N416, a child monkey of the same age (2 years old), and an aged monkey (28 years old) were taken with a 0.3 T apparatus (Vento, Hitachi). The monkeys were anesthetized with ketamine hydrochloride (5 mg/kg b.wt., i.m.) and medetomidine (100 µg/kg b.wt., i.m.), followed by head positioning with the stereotaxic apparatus [5].

### Nerve conduction velocity

To assess the functional viability of the peripheral nervous system, the conduction velocity of sensory and motor nerves was measured from the ulnar nerve of monkey N416, in comparison with those in control Japanese monkeys including 5 child monkeys (Child; 2 years old), 5 adult monkeys (Adult; 5–8 years old), and 5 aged monkeys (Aged; 25–29 years old). Measurements were done with NeuroPak (Nihon Kohden, Tokyo). The monkeys were sedated with ketamine hydrochloride (2.5 mg/kg) and medetomidine (100 µg/kg), and anaesthetized with 2% sevoflurane during the measurement. The room temperature was kept at 20°C. To measure the sensory conduction velocity (SCV), pairs of recording surface electrodes were placed over the ulnar nerve 2–3 cm proximal to the elbow and 2–3 cm proximal to the wrist, and supramaximal stimuli were delivered through a pair of ring electrodes attached to the distal and proximal interphalangeal joints of the little finger. To measure the motor conduction velocity (MCV), recording surface electrodes were placed over the abductor digiti minimi and the ulnar nerve was stimulated 2–3 cm proximal to the elbow and 2–3 cm proximal to the wrist, with supramaximal amplitude.

### Measurements of blood and urinary biomarkers

Blood and urine samples were obtained from monkey N416 (2 years and 6 months old), 5 child monkeys (age-matched), 5 adult monkeys (6–9 years old), and 5 aged monkeys (21–27 years old). Measurements of hemoglobin A1c (HbA1c; blood), hyaluronan (urine and serum), glucose (urine and serum), low-density lipoproteins (LDL; serum), high-density lipoproteins (HDL; serum), and triglyceride (TG; serum) were carried out by a commercial test company (Falco Biosystems).

### Statistics

Student's t-test was applied for cell proliferation time comparison. One-way ANOVA and *post hoc* test (Bonferroni) were applied for analyses of the ratio of senescent cells, the number of AP sites, the levels of biomarkers, and the conduction velocity of peripheral nerves (Kaleidagraph 4.1, Hulinks, Japan). Linear discriminant analysis was also applied for the peripheral nerve conduction velocity.

### DNA sequence

The sequences of candidate genes for progeroid syndromes, RECQ3 (Werner syndrome), RECQL4 (Rothmund-Thomson syndrome), RECQ2 (Bloom syndrome), and LMNA (Hutchinson-Gilford progeria syndrome), were determined by standard PCR and the direct sequence protocol with ExTaq DNA polymerase (Takara Bio Inc., Shiga, Japan) and BigDye Terminator v3.1 Cycle Sequencing Kit with a 3130 Genetic Analyzer (Applied Biosystems, CA, USA). Primers used in PCR are shown in Table S1. Furthermore, to identify possible genetic causes specifically occurring in monkey N416, broader candidate gene approaches were performed in 100 macaque monkeys including monkey N416 with Next Generation Sequencer (NGS) of HiSeq2000 (Illumina Inc., CA, USA). The candidates for progeroid-related genes were listed in Table S2. Genomic DNA samples were obtained from 100 macaque monkey blood samples including monkey N416. Each library for the NGS analysis was constructed by using the standard Illumina TruSeq DNA Library Prep protocol.

## Results

### Physical appearance

**Figure 1:**
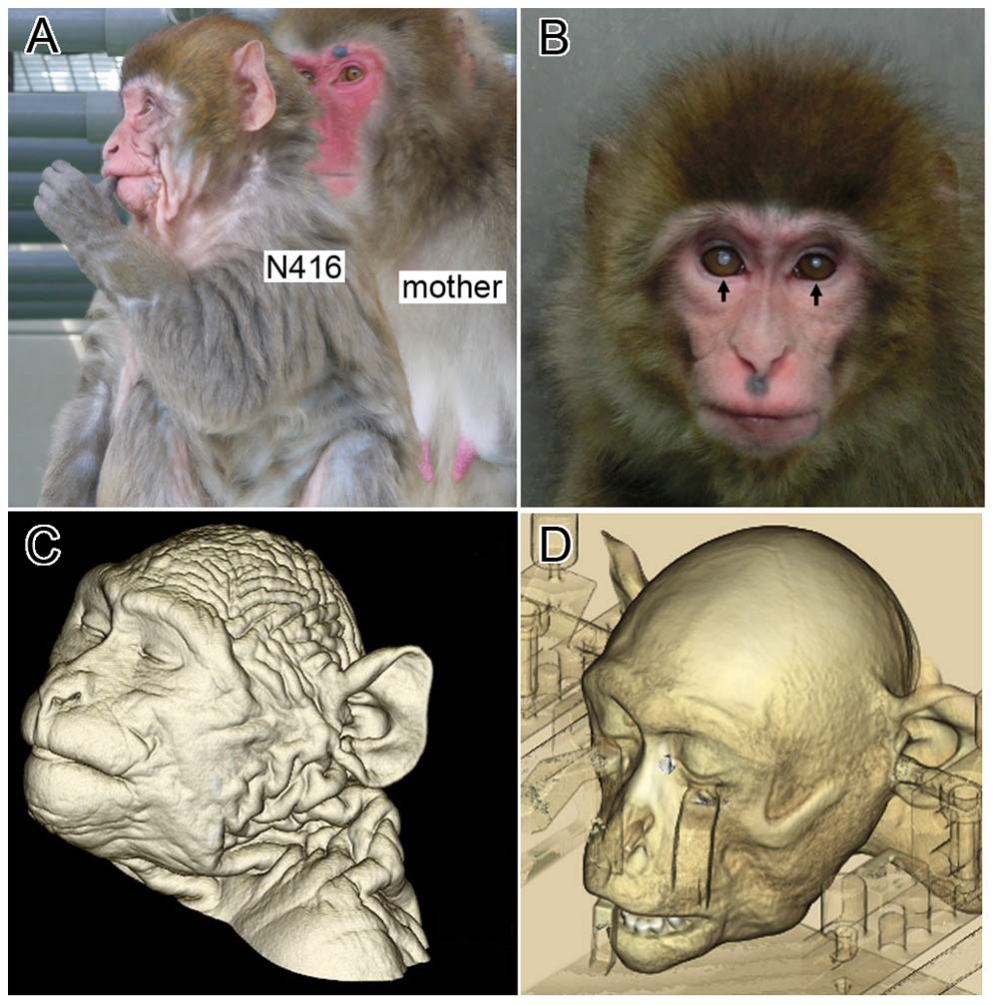
Monkey N416 resembled aged monkeys in appearance. A: Monkey N416 (10 months old) and her mother. Note that deep wrinkles of the skin are seen in monkey N416. B: Monkey N416 at the age of 1 year and 10 months. Cataract was first recognized in one eye when she was 10 months old and, progressed bilaterally within a few months (pointed to by arrows). The deep skin wrinkles become rather reduced as the body grows. C: Three-dimensionally-reconstructed CT image of monkey N416 (2 years and 5 months old). Note that the deep skin wrinkles clearly remain in both the scalp and the postcranial skin. D: Three-dimensionally-reconstructed CT image of an age-matched control. In this monkey, the skin is smooth. Semi-transparent objects are parts of the stereotaxic apparatus.

Two major appearances characteristic of monkey N416 were deep wrinkles of the skin and cataract on bilateral sides. The skin wrinkles have been eminent since she was a baby, while such wrinkles were not observed in her mother (Fig. 1A). As monkey N416 grew up, her wrinkles seemed to gradually decrease but still remained at the age of two years (Fig. 1B). Indeed, X-ray CT clearly revealed the wrinkled skin of this monkey (Fig. 1C; imaged later than the time when Fig. 1B was taken) and, as a control, the smooth skin of an age-matched normal monkey (Fig. 1D). There was no sign of ulceration of the skin, and hyperkeratosis was slightly observed in the extremities. Cataract occurred in one eye as early as monkey N416 was 9 months old, and progressed bilaterally as she grew around two years old (Fig. 1B). Based on the fact that this monkey usually reached for primate pellets in a box attached to her own cage and smelled them before eating, it appeared that her vision was still left but was only weak. When we examined her under anesthesia, we could not find out either rigidity or hyperflexibility of the limbs. No skeletal anomaly, osteoporosis, or dental crowding was found in monkey N416.

### Fibroblast cell cycle

**Figure 2:**
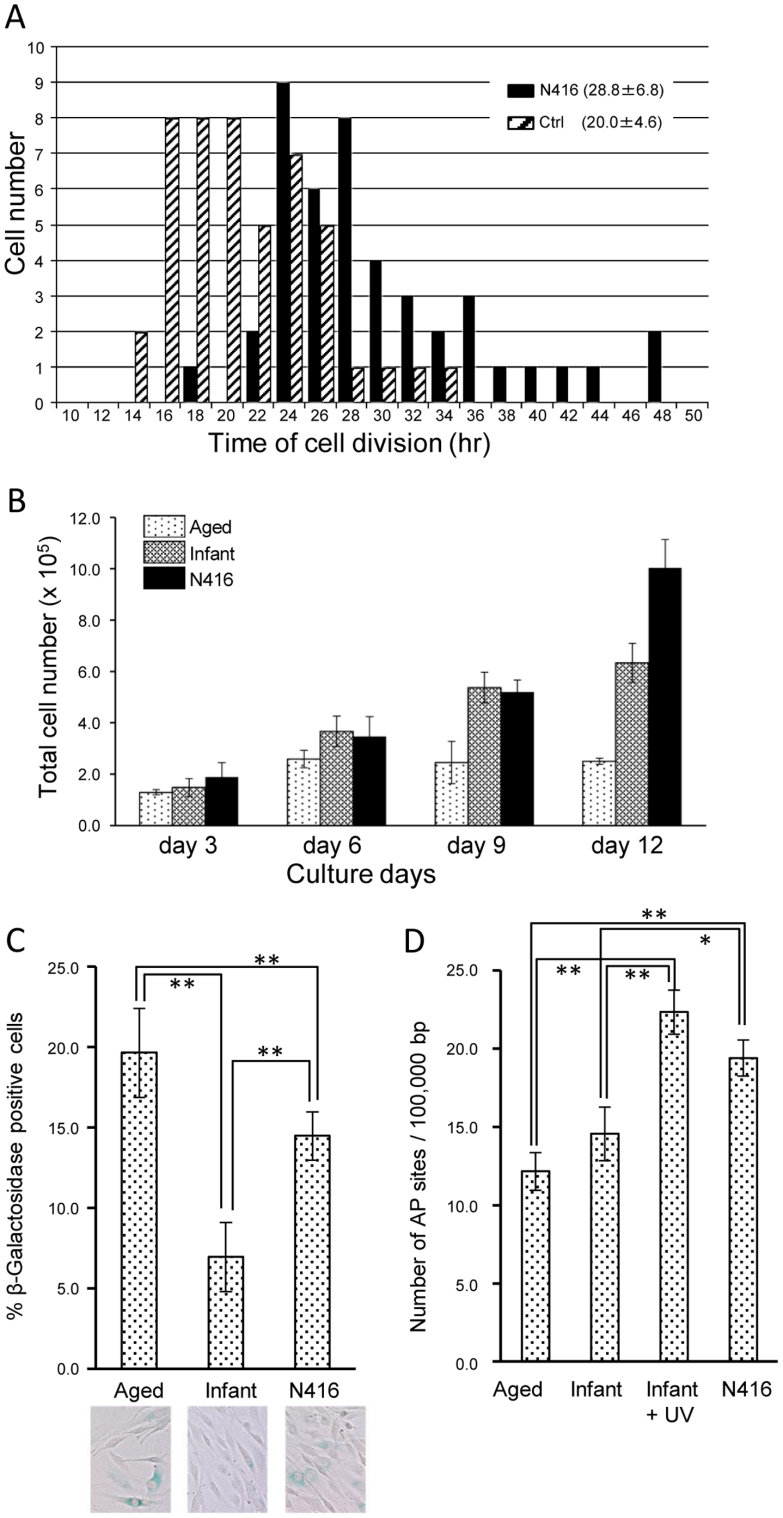
Fibroblast cells derived from monkey N416 resembled those derived from progeroid patients in cell biological features. A: Histograms of cell division intervals of fibroblasts derived from monkey N416 and a control monkey (Ctrl) at passage 5. Note that the time of cell division is significantly longer in monkey N416 (28.8±6.6 hr; n = 44) than in the control (20.0±4.6 hr, n = 47). B: Population growth of fibroblast cells derived from a normal aged monkey, a normal infant monkey, and monkey N416 at passage 8. C: Ratio of senescent-associated ß-galactosidase-positive fibroblast cells derived from monkey N416, in comparison with those derived from aged and infant monkeys (**: p<0.01). Photographs are representative images. D: Number of apurinic/apyrimidinic (AP) sites per 100,000 bp in fibroblast cells derived from monkey N416, in comparison with those from aged and infant monkeys and, also, UV-irradiated cells from the infant monkey (*: p<0.05, **: p<0.01).

Our time-lapse video analysis at passage 5 confirmed that the time for fibroblast cell cycle was significantly longer in the model monkey N416 than in a normal control (Fig. 2A; N416 28.8±6.8 hr, control 20.0±4.6 hr, t = −7.17, p<0.01). Population growth of fibroblast cells at passage 8 from the aged monkey reached a plateau at day 6. On the other hand, population growth of fibroblast cells from monkey N416 and the infant monkey continued up to day 12 (Fig. 2B).

### Senescent cells

Cytochemical assay of ß-galactosidase activity revealed that the ratio of senescent (ß-galactosidase-positive) fibroblast cells was higher in the aged monkey than in the infant monkey. Their ratio in monkey N416 was significantly lower than in the aged monkey and higher than in the infant monkey (Fig. 2C; aged 19.6±2.8%, infant 7.0±2.2%, N416 14.5±1.5%, F(2,12) = 41.9, p<0.001, one-way ANOVA; N416 vs. aged, N416 vs. infant, p<0.01, post hoc test).

### DNA repair

Deficiency of DNA repair was estimated through AP site quantification of DNA extracted from fibroblast cells. One-way ANOVA and host hoc test revealed that numbers of AP sites were higher in cells from monkey N416 and UV-irradiated cells from the infant monkey than in cells from the aged and infant monkeys (Fig. 2D; aged 12.2±1.2, infant 14.6±1.7, UV-irradiated infant 22.3±1.4, N416 19.4±1.2, F(3,8) = 33.2, p<0.001, one-way ANOVA; N416 vs. aged, UV-irradiated infant vs. aged, UV-irradiated infant vs. infant, p<0.01, N416 vs. infant, p<0.05, post hoc test).

### Blood and urinary biomarkers

**Figure 3:**
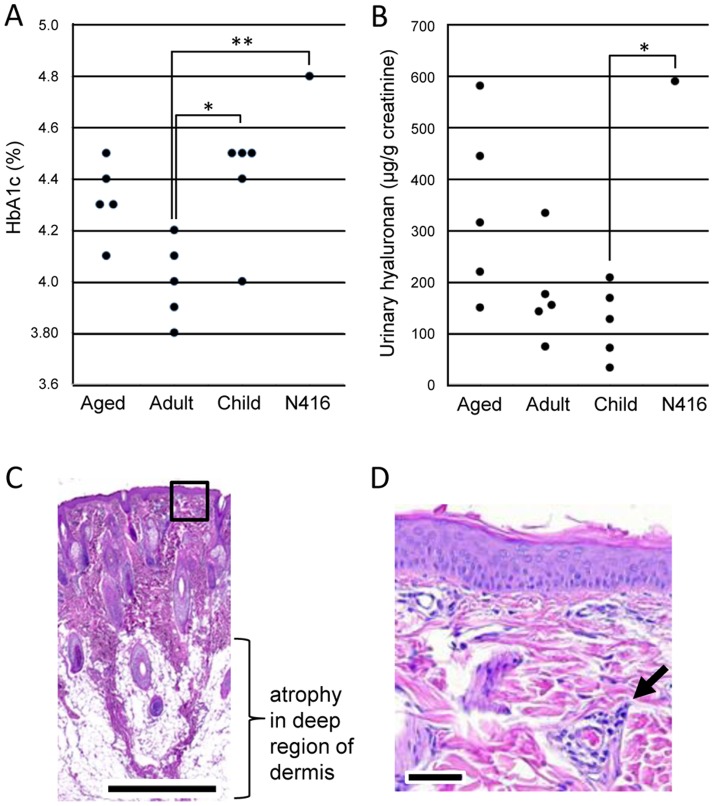
Monkey N416 resembled progeroid patients in metabolic and dermatological features. A,B: Levels of blood HbA1c (A) and urinary hyaluronan (B) in monkey N416 and the aged, adult, and child monkey groups. A significant difference between monkey N416 and the adult monkey group or between monkey N416 and the child monkey group is indicated (*: p<0.05, **: p<0.01). C,D: Poikiloderma with superficial telangiectasia of the skin in monkey N416. Atrophy in dermis is observed in C, while slight acanthosis in epidermis and expanded capillary in the superficial region of dermis (arrow) are seen in D (specified by the box in C). Bars, 1 mm in C and 100 µm in D.

We measured levels of blood and urinary biomarkers in monkey N416 and compared these values with those in aged, adult, and child monkeys. HbA1c in monkey N416 was not different from those in the aged and child groups (Fig. 3A), but was significantly higher than that in the adult group (N416 4.8%, aged 4.1–4.5%, adult 3.8–4.2%, child 4.0–4.5%, F(3, 12) = 7.642, p<0.01, one-way ANOVA; N416 vs. adult, p<0.01, post hoc test). Hyaluronan in urine in monkey N416 was not different from those in the aged and adult groups (Fig. 3B), but was significantly higher than that in the child group (N416 590 µg/g creatinine, aged 148–582 µg/g, adult 72–333 µg/g, child 31–207 µg/g, F(3, 12) = 5.938, p<0.05, one-way ANOVA; N416 vs. child, p<0.05, post hoc test). There was no significant difference in levels of hyaluronan in serum, glucose in serum and urine, LDL, HDL, or TG between monkey N416 and the other monkey groups (see Table S3).

### Pathological features

Pathological examination of the skin showed slight acanthosis and slight hypertrophy in epidermis, atrophy in dermis, and normal feature in subcutis (Fig. 3C). Angiectasia in the superficial vascular plexus of dermis, and slight infiltration of monocular cells and eosinophils in the superficial perivascular region of dermis were also observed (Fig. 3D).

According to autopsy, slight hypertrophy of the heart was found, but no signs of atherosclerosis or malignant tumors were seen. Chronic pyelonephritis in the kidney was observed as another pathological change, except for congestion in the lung, the liver, and the stomach that was caused by bloat.

### Brain structure

**Figure 4:**
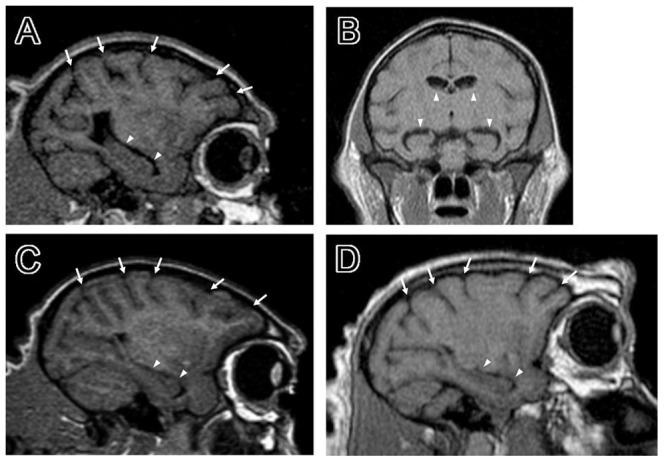
Shrinkage of the cerebral cortex and the hippocampus in monkey N416 was revealed with MRI. A,C,D: Parasagittal images of monkey N416 (1 year and 5 months old), an age-matched control, and an aged monkey (28 years old), respectively. Note that expansion of the cerebral sulci (arrows) in monkey N416 is as large as and expansion of the lateral ventricles (arrowheads) is larger than those in the aged monkey. This is probably ascribed to shrinkage of the cerebral cortex and the hippocampus. B: Coronal image of monkey N416. Seen is prominent expansion of the lateral ventricles (arrowheads).

Shrinkage of the cerebral cortex and the hippocampus was markedly observed in monkey N416, although the size of her brain was not different from that of the control child monkey brain. Consequently, the cerebral sulci and lateral ventricles were largely expanded (Fig. 4A,B), as compared to an age-matched child monkey (Fig. 4C). The same feature was seen in an aged monkey (Fig. 4D).

### Conduction velocity of peripheral nerves

**Figure 5:**
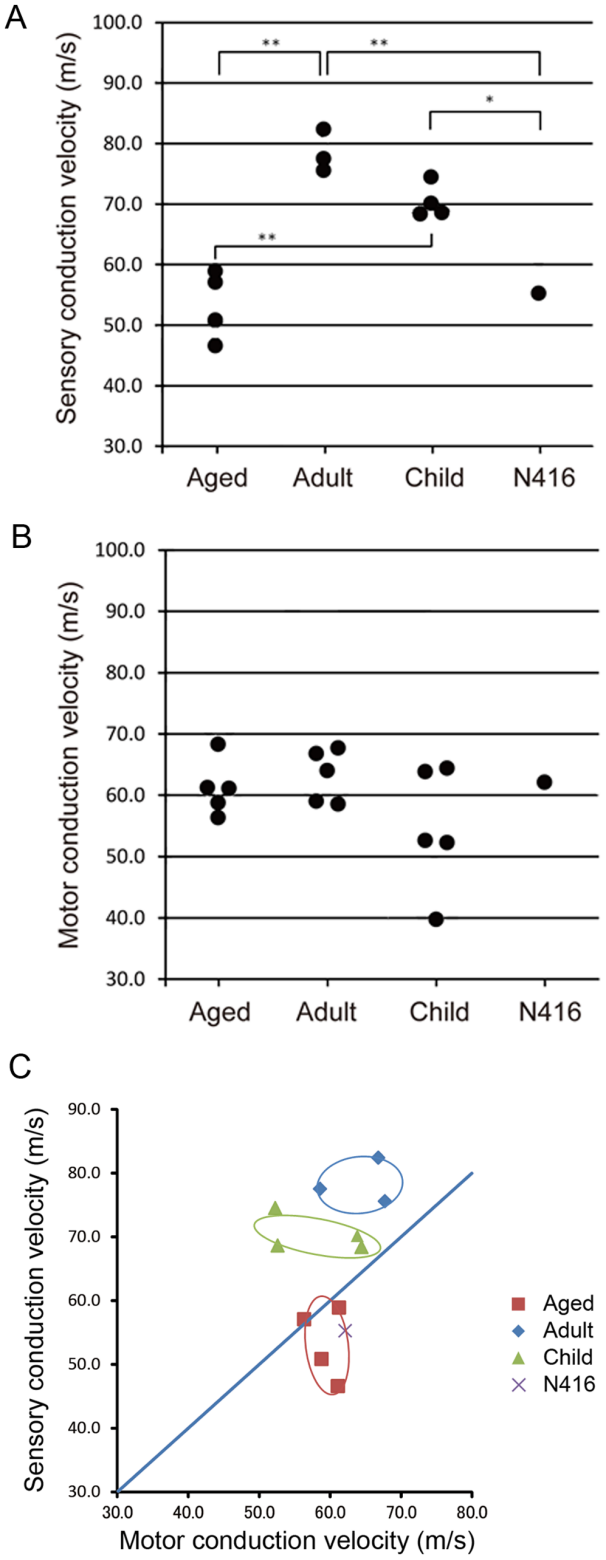
Conduction velocity of peripheral sensory but not motor nerve was slower in monkey N416. Measured from the ulnar nerve. A: Sensory conduction velocity (SCV). The SCV in monkey N416 and aged monkeys was slower than that in adult and child monkeys. **: p<0.01, *: p<0.05 (one-way ANOVA and Bonferroni *post hoc* test). B: Motor conduction velocity (MCV). The MCV in monkey N416 was similar to that in aged, adult, and child monkeys. C: SCV versus MCV in individual monkeys. Ovals indicate 1.5 standard deviation limits. Note that each age group makes a cluster, and that the position for monkey N416 is located within the cluster for the aged monkey group.

The conduction velocity of peripheral sensory and motor nerves was measured from the ulnar nerve. The sensory conduction velocity (SCV) in monkey N416 was compared with that in aged, adult, and child monkeys; each value was 55.2, 53.4±5.7 (n = 4), 78.5±3.5 (n = 3), and 70.4±2.8 (n = 4) ms, respectively (Fig. 5A). It was revealed that the SCV in monkey N416 and the aged group was significantly slower than in the adult and child groups (one-way ANOVA and post hoc test). Likewise, the motor conduction velocity (MCV) in monkey N416 was examined in comparison with that in the aged, adult, and child groups; each value was 62.1, 61.2±4.5 (n = 5), 63.2±4.3 (n = 5), and 57.0±13.8 (n = 5) ms, respectively (Fig. 5B). Thus, the MCV in monkey N416 was not significantly different from that in the aged, adult, or child monkey group (one-way ANOVA). Scattergram of SCV and MCV shows that data points for the aged, adult, and child monkeys appeared to constitute three separate clusters, and the data point for monkey N416 was included within the cluster for the aged group (Fig. 5C).

### DNA sequences of progeroid-related genes

The sequences of progeroid-related genes, RECQ3, RECQL4, RECQ2, and LMNA, were determined in monkey N416 and normal Japanese monkeys (n = 7, 41, 2, and 4, respectively) and a normal rhesus monkey, as referenced with those of rhesus monkey genomic sequence (rheMac2). Though there were five non-synonymous point mutations in recQL4 gene of monkey N416 with reference to rhesus sequences (exon3 183A>G, 387C>T, exon4 157G>A, 208T>C, 315C>T), such mutations were shared by 14 normal monkeys. Thus, these mutations were not symptom-specific. The sequences of RECQ3, RECQ2 and LMNA gene in monkey N416 were identical to that of rhesus monkey.

To further extend the DNA sequencing analysis, we adapted a large scale of DNA sequencing approaches using a next generation sequencer (NGS). Specifically, 21 candidates of progeroid-related genes were sequenced in 100 monkeys including monkey N416 (see Table S2). The results showed that two genes (NBN and DDB2) have N416-specific mutations out of the 100 monkeys. However, these mutations are synonymous substitutions in NBN genes and downstream mutations in DDB2 genes, respectively, suggesting that an impact of such mutations on phenotypic functions is thought to be very weak.

## Discussion

In our institute, we found a child Japanese monkey (monkey N416) who exhibited deep skin wrinkles and bilateral cataract within one year after birth. The following abnormalities were also found in this monkey: retardation of fibroblast proliferation at passage 5, appearance of senescent cells, deficiency in DNA repair, impaired glucose metabolism, altered hyaluronan metabolism, poikiloderma, shrinkage of the cerebral cortex and the hippocampus, and decrease in the conduction velocity of peripheral sensory nerves. However, the growth of this monkey was not retarded before she was 3 years old. These findings suggest that monkey N416 may probably suffer from a certain type of progeria.

### Cataract

Bilateral cataract in monkey N416 seemed severe. In an individual cage, this monkey frequently smelled food pellets. As normal monkeys do not smell ordinary food so frequently, such a behavior may result from weak vision caused by cataract.

### Retardation of fibroblast proliferation

Retardation of fibroblast proliferation is characteristic of WS and HGPS [6], [7]. Our cell-culture examination showed a prolonged duration for fibroblast proliferation at passage 5 in monkey N416 as compared to a normal control, at a single cell level. This suggests that this monkey is likely progeroid. At passages 8, on the other hand, the population growth activity in fibroblast cells derived from monkey N416 was as high as that from the infant monkey, and higher than that from the aged monkey. This discrepancy can be accounted for by postulating that the difference in the manner of fibroblast proliferation in these experiments may be attributed to the difference in the passage number: passage 5 cells used for single-cell level analysis vs. passage 8 cells used for population level analysis. To address this issue, further investigations, for example flow cytometry of cell cycle constitution in early and late cultures, are needed.

### Appearance of senescent cells

Acidic ß-galactosidase is known to be a good hallmark of senescent cells [8]. Fibroblasts from HGPS patients express strong ß-galactosidase activity [9]. Though it was previously reported that oxydative stress did not increase senescence-associated ß-galactosidase activity in fibroblasts with WS [10], knockdown of RECQ2, RECQ3, and RECQL4 resulted in an increase in ß-galactosidase-positive fibroblasts [11]. Our cytochemical analysis showed that the ratio of ß-galactosidase-positive fibroblasts in monkey N416 was lower than in the aged monkey, and higher than in the infant monkey. Considering the difference in the number of passage of fibroblasts (N416 and aged 5, infant 7) and the age (infant 0 year, N416 3 years, aged 21 years), the ratio of senescent fibroblast cells in monkey N416 could be reasonable as a result of aging.

### Deficiency in DNA repair

DNA repair deficiency is a common feature of HGPS [12] and other progeroid syndromes. RECQ3, RECQL4, and RECQ2 encode helicases, involved in DNA repair [1]. Our AP site assay have revealed that DNA repair does not simply deteriorate with the age, and that clear deficiency in DNA repair is observed in monkey N416. These findings strongly support that monkey N416 may suffer from a type of progeria.

### Impaired glucose metabolism

It is well known that diabetes mellitus (DM) develops in most patients with WS [13]. Thus, we examined the stable DM marker, HbA1c. Levels of HbA1c in aged, adult, and child monkeys were as high as that in healthy rhesus (<4.7) [14] and long-tailed (4.4±0.1) [15] monkeys. However, HbA1c in monkey N416 was 4.8, estimated as “glucose impaired”, and significantly higher than in adult monkeys. This suggests that monkey N416 exhibits glucose metabolism impairment in accordance with WS patients, though she has not developed DM yet.

### Altered hyaluronan metabolism

Hyaluronan is a key molecule to shape skin characteristics. For example, the Shar-pei dog and human patients with similar abnormal deep skin wrinkles share impairment in hyaluronan metabolism. Skin wrinkles in the Shar-pei dog results from upregulation of hyaluronic acid synthase 2 [16]. Deep skin wrinkles seen in human patients are correlated with an elevated hyaluronan concentration in serum [17]. Also, the hyaluronan level in WS is relatively high in urine [18]. The urinary hyaluronan level in monkey N416 was significantly higher than in child monkeys and was located between human WS patients and healthy controls [18]. The urinary hyaluronan level increased with the age in Japanese monkeys like humans, though the level itself was lower [18].

### Poikiloderma and other chronic pathological changes

Based on the present pathological findings on the skin (slight acanthosis in epidermis, atrophy in dermis, and normal feature in subcutis, angiectasia in the superficial vascular plexus of dermis, and slight cellular infiltration of monocular cell and eosinophils in the superficial perivascular region of dermis), it can be considered that monkey N416 has poikiloderma with superficial telangiectasia. As poikiloderma is a representative feature of RTS, and BS [1], [19], this favors that such pathological features may be ascribable to progeria.

In addition, there were chronic pathological changes in monkey N416, such as slight cardiac hypertrophy and renal pyelonephritis. It should be emphasized here, however, that these are quite rare in normal child monkeys.

### Nervous systems

Expansion of the cerebral sulci and lateral ventricles, due to shrinkage of the cerebral cortex and the hippocampus, is a prominent aging-related change in the central nervous system of humans and rhesus monkeys [20]. In the present study, our MRI analysis indicated that the same event occurred in monkey N416 as well as in an aged control, but not in a child control. It remains to be known whether there are any histological changes (i.e., neuronal degeneration/loss) or molecular events that may be caused by aging.

The conduction velocity of peripheral sensory and motor nerves decreases when demyelination progresses [21]. In our experiments, we analyzed the conduction velocity of the ulnar nerve in monkey N416 by comparing with those in aged, adult, and child monkeys. The sensory conduction velocity (SCV) was significantly lower in monkey N416 and the aged monkey group than in the adult and child monkey groups. The SCV in adult and child monkeys was similar to that reported in the baboon [22]. Thus, it is most likely that demyelination of the sensory nerve may occur not only in the aged monkeys, but also in monkey N416. By contrast, the motor conduction velocity (MCV) in monkey N416 was not different from that in aged, adult, or child monkeys. The MCV measured in our study was similar to that reported in rhesus and long-tailed monkeys [23], [24]. Further investigations are needed to clarify whether demyelination of the sensory nerve may occur in monkey N416 and whether the discrepancy between the SCV and the MCV may be ascribed to demyelination.

### Progeroid-related genes

No individual-unique mutations of known progeroid-related genes were found in monkey N416. Some point mutations with reference to rhesus sequences were shared with normal monkeys. Therefore, it can be considered that phenotypes of monkey N416 are not simply attributed to such point mutations. To survey candidate genes responsible for progeroid phenotypes observed in monkey N416, further intensive analyses of the whole genome, at least at the exome level, are indispensable.

### Comparison with major progeroid syndromes

As described above, monkey N416 displayed eminent symptoms of premature aging, such as bilateral cataract, retardation of fibroblast proliferation, appearance of senescent cells, deficiency in DNA repair, impaired glucose metabolism, altered hyaluronan metabolism, and poikiloderma. However, we could not detect any individual-unique mutations of known genes responsible for major progeroid syndromes. Typical facial features of HGPS include a small jaw, loss of hair, and scalp veins [2], none of which were observed in monkey N416. Moreover, this monkey did not show growth impairment, sclerotic skin, or joint contractures, each of which is characteristic of HGPS [25]. RTS is characterized by juvenile cataract, skeletal abnormality, proportionate short stature, and poikiloderma [1]. Juvenile cataract and poikiloderma were the common feature in both monkey N416 and RTS. WS is the most popular progeroid syndrome, though its frequency is less than 1/100,000 [26]. Some symptoms in monkey N416 were consistent with those in WS, including bilateral cataract, increased urinary hyaluronan, increased HbA1c, and retarded cell proliferation [18]. However, many symptoms seen in WS emerge only after adolescence [13], whereas several symptoms appear as early as 1 year old in monkey N416. In addition, there was no sign of osteoporosis, ulceration or atrophy of the skin, or calcification of the Achilles tendon [27], suggesting that monkey N416 does not suffer from WS. Progeroid phenotypes manifested in monkey N416 are similar to in some aspects, but rather different from those in such major progeroid syndromes in humans (see Table 1).

In conclusion, the present study is the first to report a naturally occurring primate model with sporadic symptoms of premature aging. Further investigations especially with genome analyses and cell cultures will help us understand normal aging as well as progeria in primates including humans.

## Supporting Information

Table S1 **Sequence of primers used in PCR.**
(XLS)

Table S2 **Candidates for progeroid-related genes.** There were N416 specific mutations in two progeria-related genes (NBN and DDB2). In the NBN, heterozygous synonymous mutation was found at exon 4, positioning of chr8:92,628,839 (coordinated by rheMac2), and in the DDB2, homozygous mutation was found at the downstream region, positioning of chr14:24,879,090 (also coordinated by rheMac2).
(XLS)

Table S3 **Levels of biomarkers in serum and urine, which showed no difference among N416 and age groups.**
(XLS)

## References

[1] Mohaghegh P, Hickson ID (2002) Premature aging in RecQ helicase-deficient human syndromes. Int J Biochem Cell Biol 34: 1496–1501. 12200042 10.1016/s1357-2725(02)00039-0

[2] Pollex RL, Hegele RA (2004) Hutchinson-Gilford progeria syndrome. Clin Genet 66: 375–381. 15479179 10.1111/j.1399-0004.2004.00315.x

[3] Hoki Y, Araki R, Fujimori A, Ohhata T, Koseki H, et al (2003) Growth retardation and skin abnormalities of the Recql4-deficient mouse. Hum Mol Genet 12: 2293–2299. 12915449 10.1093/hmg/ddg254

[4] Hirai H, Hirai Y, Kawamoto Y, Endo H, Kimura J, et al (2002) Cytogenetic differentiation of two sympatric tree shrew taxa found in the southern part of the Isthmus of Kra. Chrom Res 9: 313–327. 10.1023/a:1016523909096 12199145

[5] Nishimura T, Oishi T, Suzuki J, Matsuda K, Takahashi T (2008) Development of the supralaryngeal vocal tract in Japanese macaques: implications for the evolution of the descent of the larynx. Am J Phys Anthropol 135: 182–194. 17960727 10.1002/ajpa.20719

[6] Epstein C, Martin G, Motulsky A (1965) Werner's syndrome; caricature of aging. A genetic model for the study of degenerative diseases. Trans Assoc Am Physicians 78: 73–81. 5864984

[7] Danes BS (1971) Progeria: a cell culture study on aging. J Clin Invest 50: 2000–2003. 5564400 10.1172/JCI106692 PMC292126

[8] Dimri GP, Lee X, Basile G, Acosta M, Scott G, et al (1995) A biomarker that identifies senescent human cells in culture and in aging skin *in vivo* . Proc Natl Acad Sci U S A 92: 9363–7. 7568133 10.1073/pnas.92.20.9363 PMC40985

[9] Gordon LB, Cao K, Collins FS (2012) Progeria: translational insights from cell biology. J Cell Biol 199: 9–13. 23027899 10.1083/jcb.201207072 PMC3461511

[10] de Magalhães JP, Migeot V, Mainfroid V, de Longueville F, Remacle J, et al (2004) No increase in senescence-associated beta-galactosidase activity in Werner syndrome fibroblasts after exposure to H_2_O_2_ . Ann N Y Acad Sci 1019: 375–8. 15247048 10.1196/annals.1297.066

[11] Lu H, Fang EF, Sykora P, Kulikowicz T, Zhang Y, et al (2014) Senescence induced by RECQL4 dysfunction contributes to Rothmund-Thomson syndrome features in mice. Cell Death Dis 5: e1226. 24832598 10.1038/cddis.2014.168 PMC4047874

[12] Epstein J, Williams JR, Little JB (1973) Deficient DNA repair in human progeroid cells. Proc Natl Acad Sci U S A 70: 977–81. 4515628 10.1073/pnas.70.4.977 PMC433405

[13] Goto M (1997) Hierarchical deterioration of body systems in Werner's syndrome: Implications for normal ageing. Mechanisms of Ageing and Development 98: 239–254. 9352493 10.1016/s0047-6374(97)00111-5

[14] McTighe MS, Hansen BC, Ely JJ, Lee DR (2011) Determination of hemoglobin A1c and fasting blood glucose reference intervals in captive chimpanzees (Pan troglodytes). J Am Assoc Lab Anim Sci 50: 165–170. 21439208 PMC3061415

[15] Marigliano M, Casu A, Bertera S, Trucco M, Bottino R (2011) Hemoglobin A1C Percentage in Nonhuman Primates: A Useful Tool to Monitor Diabetes before and after Porcine Pancreatic Islet Xenotransplantation. J Transplant 2011: 965605. 21559266 10.1155/2011/965605 PMC3087943

[16] Olsson M, Meadows J, Truvé K, Rosengren Pielberg G, Puppo F, et al (2011) A novel unstable duplication upstream of HAS2 predisposes to a breed-defining skin phenotype and a periodic fever syndrome in Chinese Shar-Pei dogs. PLos Genetics 7: e1001332. 21437276 10.1371/journal.pgen.1001332 PMC3060080

[17] Ramsden CA, Bankier A, Brown TJ, Cowen PS, Frost GI, et al (2000) A new disorder of hyaluronan metabolism associated with generalized folding and thickening of the skin. J Pediatr 136: 62–68. 10636976 10.1016/s0022-3476(00)90051-9

[18] Tanabe M, Goto M (2001) Elevation of serum hyaluronan level in Werner's syndrome. Gerontology 47: 77–81. 11287731 10.1159/000052777

[19] Arora H, Chacon AH, Choudhary S, McLeod MP, Meshkov L, et al (2014) Bloom syndrome. Int J Dermatol 53: 798–802. 24602044 10.1111/ijd.12408

[20] Peters A, Rosene DL (2003) In aging, is it gray or white? J Comp Neurol 462: 139–143. 12794738 10.1002/cne.10715

[21] McDonald WI (1963) The effects of experimental demyelination on conduction in peripheral nerve: a histological and electrophysiological study. II. Electrophysiological observations. Brain 86: 501–524. 14063896 10.1093/brain/86.3.501

[22] Hopkins AP, Gilliatt RW (1971) Motor and sensory nerve conduction velocity in the baboon: normal values and changes during acrylamide neuropathy. J Neurol Neurosurg Psychiatry 34: 415–426. 4328885 10.1136/jnnp.34.4.415 PMC493816

[23] Purser DA, Berrill KR, Majeed SK (1983) Effects of lead exposure on peripheral nerve in the cynomolgus monkey. Br J Ind Med 40: 402–412. 6626468 10.1136/oem.40.4.402 PMC1009213

[24] Weimer MB, Gutierrez A, Baskin GB, Borda JT, Veazey RS, et al (2005) Serial electrophysiologic studies in rhesus monkeys with Krabbe disease. Muscle Nerve 32: 185–190. 15937878 10.1002/mus.20350

[25] Merideth MA, Gordon LB, Clauss S, Sachdev V, Smith AC, et al (2008) Phenotype and course of Hutchinson-Gilford progeria syndrome. N Engl J Med 358: 592–604. 18256394 10.1056/NEJMoa0706898 PMC2940940

[26] Goto M (2004) Clinical Aspects of Werner's Syndrome: Its Natural History and the Genetics of the Disease. In: M L, editor. Molecular Mechanisms of Werner's Syndrome. New York: Kluver Academic Plenum Publishers. pp. 1–11.

[27] Takemoto M, Mori S, Kuzuya M, Yoshimoto S, Shimamoto A, et al (2013) Diagnostic criteria for Werner syndrome based on Japanese nationwide epidemiological survey. Geriatr Gerontol Int 13: 475–481. 22817610 10.1111/j.1447-0594.2012.00913.x

